# Hepatitis B and C virus infection among HIV patients within the public and private healthcare systems in Chile: A cross-sectional serosurvey

**DOI:** 10.1371/journal.pone.0227776

**Published:** 2020-01-09

**Authors:** Thomas Weitzel, Fernanda Rodríguez, Luis Miguel Noriega, Alejandra Marcotti, Luisa Duran, Carla Palavecino, Lorena Porte, Ximena Aguilera, Marcelo Wolff, Claudia P. Cortes

**Affiliations:** 1 Laboratorio Clínico, Clínica Alemana de Santiago, Facultad de Medicina Clínica Alemana, Universidad del Desarrollo, Santiago, Chile; 2 Instituto de Ciencias e Innovación en Medicina (ICIM), Facultad de Medicina Clínica Alemana, Universidad del Desarrollo, Santiago, Chile; 3 Fundación Arriarán, Santiago, Chile; 4 Departamento de Medicina Interna, Clínica Alemana de Santiago, Facultad de Medicina Clínica Alemana, Universidad del Desarrollo, Santiago, Chile; 5 Departamento de Medicina, Facultad de Medicina, Universidad de Chile, Santiago, Chile; 6 Centro de Epidemiología y Políticas de Salud, Facultad de Medicina Clínica Alemana, Universidad del Desarrollo, Santiago, Chile; Centers for Disease Control and Prevention, UNITED STATES

## Abstract

**Background:**

Coinfections of HIV patients with hepatitis B virus (HBV) and hepatitis C virus (HCV) are mayor public health problems, contributing to the emerging burden of HIV-associated hepatic mortality. Coinfection rates vary geographically, depending on various factors such as predominant transmission modes, HBV vaccination rates, and prevalence of HBV and HCV in the general population. In South America, the epidemiology of coinfections is uncertain, since systematic studies are scarce. Our study aimed to analyze rates of HBV and HCV infection in people living with HIV attending centers of the public and private health system in Chile.

**Methods:**

We performed a cross-sectional study including a public university hospital and a private health center in Santiago, Metropolitan Region in Chile. Serum samples were used to determine serological markers of hepatitis B (HBsAg, anti-HBs, anti-HBc total, HBeAg, anti-HBe) and anti-HCV. Demographic, clinical and laboratory data were obtained from medical records.

**Results:**

399 patients were included (353 from public, 46 from private health center). Most (92.8%) were male, with a median age of 38.3 years; 99.4% acquired HIV through sexual contact (75.0% MSM); 25.7% had AIDS and 90.4% were on ART. In 78.9%, viral loads were <40 cps/mL; the median CD4 cell count was 468 cells/mm^3^. According to their serological status, 37.6% of patients were HBV naïve (susceptible), 6.5% were vaccinated, 43.6% had resolved HBV infection, and 5.8% were chronically infected. The rate of vaccination was 4.5% in the public and 21.7% in the private system. HCV coinfection was found in 1.0% of all patients.

**Conclusion:**

HBV coinfection rate was within the range of other South American countries, but lower than in non-industrialized regions in Asia and Africa. A low percentage of patients were HBV vaccinated, especially within the public system. HCV coinfection rate was very low, most probably due to the rareness of injecting drug use.

## Introduction

According to UNAIDS, 71,000 Chileans aged ≥15 are infected with HIV, approximately 87% of them are aware of their HIV status, and 63% are on antiretroviral treatment (ART) [1]. The majority (78%) is covered by the public health insurance and attended at public health institutions, while only 14% rely on private health insurers [2]. The latter attend private healthcare providers, often as preferred provider organizations, depending on their insurance arrangement and financial capability. Since 2001, the Chilean Ministry of Health implemented an HIV extended access program (EAP), including free access to ART and treatment monitoring [3] The vast majority (85%) of people living with HIV (PLWH) all over Chile are attended by a network of 32 entry points within the public healthcare system, which form the Chilean HIV cohort [4, 5]. The number of PLWH covered by private health insurances was 8,200 in 2017 [6]. Patients of both systems are covered by the EAP and treated in a standardized way, resulting in high rates of treatment with long-term virological suppression [5, 7].

Coinfection of human immunodeficiency virus (HIV) with hepatitis B virus (HBV) or hepatitis C (HCV) are major public health problems worldwide. Chronic viral hepatitis has emerged as an important cause of morbidity and mortality among PLWH [8, 9], resulting in an increase in inpatient healthcare utilization and an evolving discussion on the use of liver transplantation in these patients [10, 11].

Globally, 257 million people are chronically infected with HBV[12]. Since HIV and HBV share the same transmission routes (mainly sexual), coinfection is frequent, especially within key populations including men who have sex with men (MSM)[13]. It is estimated that approximately 7.4% of PLWH are chronically HBV infected; conversely, about 1% of those with chronic HBV infection are HIV infected [14]. Coinfection rates are geographically heterogeneous and vary from 5% to up to 20% worldwide, depending on various factors such as distribution of risk groups, implementation of HBV vaccination programs, and levels of endemicity in the general population [15–17]. The highest rates are found in Asia and Africa [18], primarily affecting vulnerable populations of low-/middle-income countries [19]. In South America, the epidemiological situation is less certain, since in most countries systematic studies are scarce [20]. In Chile, the prevalence of coinfection was 6.1% in a small single center study and 8.4% in a retrospective analysis of the database of the Chilean AIDS cohort [21, 22].

The natural history of HBV is complicated by HIV coinfection. Patients have an increased risk of death and progression to liver cirrhosis [16, 18, 23, 24]. The negative impact of HIV/HBV includes higher HBV replication levels and higher risk of chronic infection or reactivations, and progress to liver cirrhosis and hepatocellular carcinoma (HCC). This results in a higher overall mortality due to liver-related but also AIDS-associated complications [16, 25–28]. The influence of HBV on the course of HIV infection and success of antiretroviral therapy (ART) is controversial. Some studies suggest a slower HIV response [29], while others report no impact on the progression to AIDS or response to ART [30–32].

Approximately 71 million people are infected with HCV worldwide [12]. The main risk factor for HCV is injecting drug use (IDU), whereas heterosexual intercourse is not a major transmission route. MSM and women infected with HIV or other sexually transmitted diseases (STDs) seem to have an increased risk of sexual HCV transmission [33, 34]. In South America, the infection rate of PLWH with HCV is highly variable, ranging from 0.7% in Venezuela to 29.9% in Argentina [35, 36]. The estimated HCV seroprevalence of the Chilean general population is 0.3% [37, 38], while epidemiological data on HIV/HCV coinfection derive from a single center, which reported a prevalence of 2.6% [21, 35]. Since the introduction of highly active ART resulted in an overall decrease of AIDS-related mortality, end stage liver disease caused by HCV has gained importance as a cause of mortality in PLWH [39]. Similar to HBV, patients with HIV/HCV coinfection progress more frequently and faster to liver fibrosis and cirrhosis and have a higher risk of HCC. In addition, HCV treatment in PLWH is less effective [23, 39].The role of HCV as a co-factor in HIV disease progression remains controversial, but some studies have found lower absolute CD4+ cell counts and reduced immunological and virological responses after ART initiation [32, 40, 41].

As stated by the ECDC, prevention, care, and treatment of HBV, HCV, and HIV should be an integrated strategy in order to reduce disease-related mortality. The fundament of such a strategy is the knowledge of the local epidemiology of these chronic infections [13]. The presented study analyzed the rates of HBV and HCV infection among adult PLWH attending HIV clinics of the public and private health system in Santiago, Chile.

## Methods

### Ethics statements

The study protocol was reviewed and approved by the Comité de Ética de la Investigación, Universidad del Desarrollo (N° 2013–02) and the Comité de Ética en Investigación en Seres Humanos, Universidad de Chile (N° 070–2012) in Santiago, Chile. All patients provided written consent and agreed to participate in the study.

### Study setting and design

**Fig 1:**
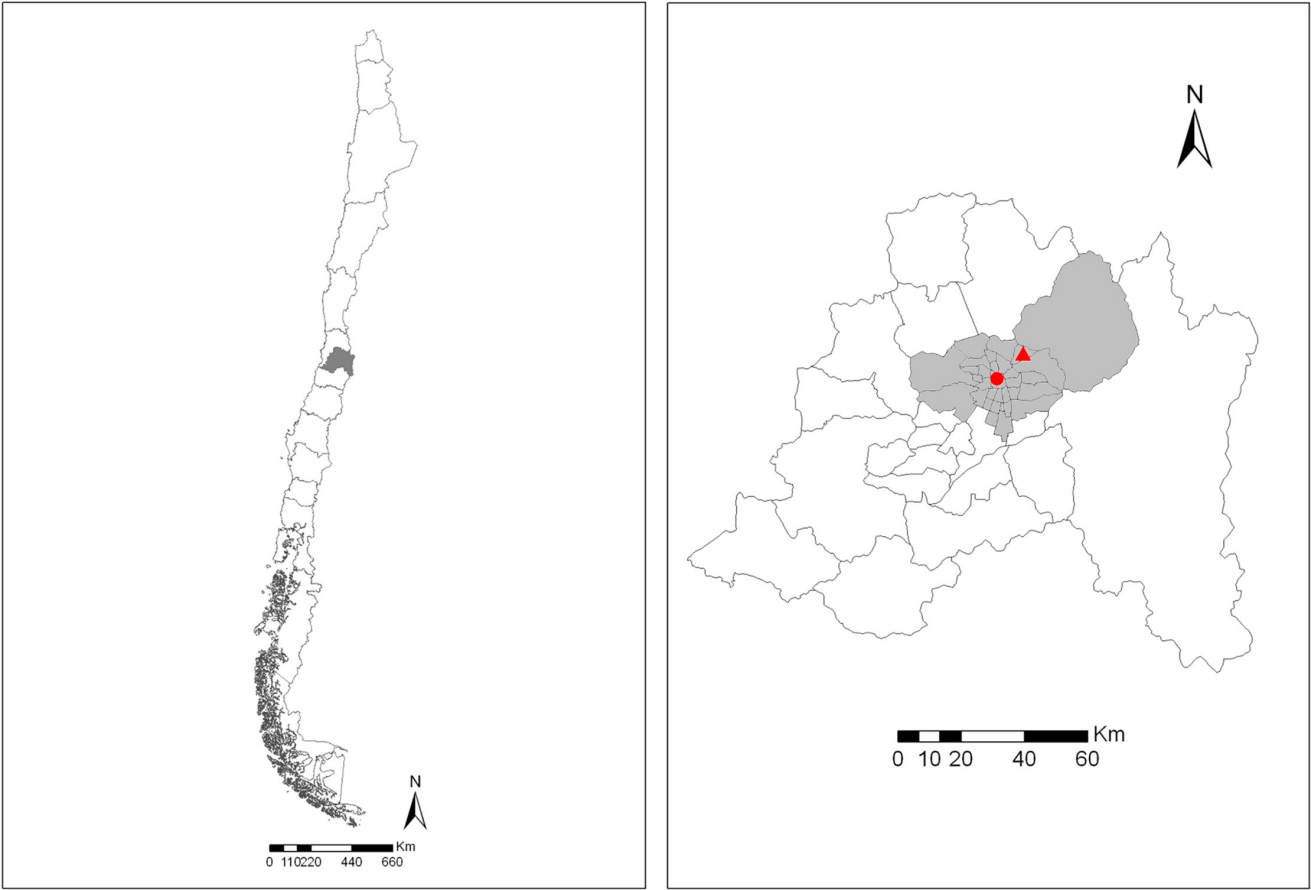
The left panel shows the Metropolitan Region (grey shading) in central Chile, where the city of Santiago is located. The right panel demonstrates the province of Santiago (grey shading) with Fundación Arriarán (red dot) located in the municipality of Santiago and Clínica Alemana (red triangle) in the Vitacura municipality.

We performed a cross-sectional study of patients attending two HIV centers: a public healthcare institution, Fundación Arriarán (Hospital Clínico San Borja Arriarán) and a private healthcare center, Clínica Alemana, both located in the Metropolitan Region of Chile (Fig 1). With 6.5 million inhabitants, this region accounts for 37% of Chile’s population. Approximately 60% of all PLWH in Chile reside in the Metropolitan Region [3].

Fundación Arriarán, located in the near of Santiago’s city center, is the oldest and leading HIV/AIDS reference center in Chile. At the time of the study, Fundación Arriarán attended approximately 3,000 PLWH, overseeing >7,000 cumulative patients. Patients live in the Metropolitan Region, mostly in the central and southern districts of Santiago. As the largest center of the Chilean HIV Cohort, it represents patients of the public health sector [42]. Clínica Alemana is a 500-bed tertiary care private medical center in one of the upper-income neighborhoods of Santiago. Together with few other private health institutions, it serves the upper-income segment of the Metropolitan Region. Its infectious diseases outpatient service attends approximately 200 PLWH (around 2% of PLWH of the private health sector).

### Sample size calculation and study population

People living with HIV, attending both health centers, age ≥ 18 years and who consented to participate in the study. Patients with a history or suspicion of acute hepatitis B during the last 6 months were excluded. The sample size was calculated based on an estimated rate of past HBV infection of 44%, a variation of 5%, power of 80% and alpha-type error of 5%, using Raosoft Sample Size Calculator (www.raosoft.com) [21]. The study population was 3,000 in Fundación Arriarán plus 200 in Clínica Alemana, and the sample size was 337 for Fundación Arriarán and 132 for Clínica Alemana.

### Data collection

Data collection was carried out from July 2014 to July 2015 on both sites. Starting in July 2014, patients who attended the study centers for control visits and who fulfilled the inclusion criteria were offered to participate in the study, until the target sample size was reached. Patients were consecutively recruited during their routine laboratory visits.

Demographic (age, sex), epidemiological (transmission) and clinical data (treatment, CD4 count, viral load) were obtained from medical records. HIV- and liver-related laboratory data within a range of 2 months from study inclusion were analyzed.

### Laboratory methods

After patients agreed to participate in the study, whole blood samples were obtained during routine laboratory controls. Serum was separated, aliquoted, and kept at -20°C prior to testing. All samples were analyzed for hepatitis B surface antigen (HBsAg), antibodies against hepatitis B surface and core antigens (anti-HBs and anti-HBc, respectively), and hepatitis C antibodies. HBsAg positive specimens were also examined for hepatitis B envelope antigen (HBeAg) and antibodies against this antigen (anti-HBe). All serological markers were determined by commercial electro-chemiluminescence immunoassays (Elecsys®, Roche Diagnostics, Mannheim, Germany) according to the manufacturer’s instructions on a Cobas e411 analyzer (Roche Diagnostics). Serological markers of hepatitis B were transferred into different profiles (Table 1) [43]. Other laboratory tests were performed within the laboratory routines of the respective centers.

**Table 1:** Serological profiles of hepatitis B infection according to serological markers.

	Serological marker				
Profile	HBsAg	anti-HBs	anti-HBc	HBeAg	anti-HBe
Naïve (susceptible)	-	-	-	NA	NA
Vaccinated	-	+	-	NA	NA
Resolved infection	-	+	+	NA	NA
Chronic infection HBeAg neg.	+	-	-	-	+
HBeAg pos.	+	-	-	+	-
Isolated Anti-HBc	-	-	+	NA	NA

HBsAg, hepatitis B virus surface antigen; anti-HBs, antibodies against HBsAg; anti-HBc, antibodies against hepatitis B virus core antigen; HBeAg, hepatitis B virus envelope antigen; anti-HBe, antibodies against HBeAg; NA, not applicable

### Outcomes

Primary outcome was the seroprevalence of hepatitis B and/or hepatitis C coinfection among the studied population of PLWH. Secondary outcomes were the prevalence values of different serological hepatitis B profiles and the demographic and clinical differences among patients from the two study sites.

### Statistical analysis

Data from clinical and serological results with their interpretation were entered into a spreadsheet (MS Excel 2013). The seroprevalence of hepatitis B and/or hepatitis C coinfections was expressed as the percentage of PLWH screened. Dichotomized and categorical data were analyzed estimating confidence interval (95%) for proportions (Wilson), using VassarStats (http://vassarstats.net).

## Results

### Patients

A total of 399 patients were tested; 353 were enrolled in the public hospital and 46 in the private center. Among them, 93% were male and the mean age was 38.3 years; 99.4% acquired HIV through sexual contact (82.7% were MSM, 16.7% were heterosexual) and 0.6% through IDU. Among the participants, 26% were staged as CDC category C and 96% were on ART. Viral load was undetectable (<40 copies/mL) in 79% and the median CD4 cell count was 499 cells/μL (IQR 349–663 cells/μL) (Table 2). The populations from the two centers did not differ regarding their demographic, clinical, and laboratory parameters with the exception that a higher percentage of patients from Fundación Arriarán had heterosexual transmission of HIV (26.4% vs. 9.8%, Table 2).

**Table 2:** Comparison of demographic, clinical, and laboratory characteristics of the participants from the two HIV centers in Santiago, Chile.

		Fundación Arriarán (n = 353)		Clínica Alemana (n = 46)		Total (n = 399)	
		Value	CI95%^a^	Value	CI95%^a^	Value	CI95%^a^
Gender	Female (%)	7.5	5.2–10.8	4.5	1.3–15.1	7.2	5.0–10.2
	Male (%)	92.5	89.2–94.8	95.5	84.9–98.7	92.8	89.8–95.0
Age	Median (years)	38.8	22.7–54.9^b^	35.9	20.0–51.8^b^	38.3	31.4–47.5^b^
Transmission	MSM (%)	73.3	68.1–77.9	87.8	74.5–94.7	75.0	70.2–79.3
	Heterosexual (%)	26.4	21.8–31.6	9.8	3.9–22.6	24.4	20.2–29.2
	IDU (%)	0.3	0.1–1.8	2.4	0.4–12.6	0.6	0.2–2.1
Clinical category [44]	A (%)	61.7	56.2–66.9	58.8	42.2–73.6	61.4	56.2–66.4
	B (%)	13.0	9.7–17.1	11.8	4.7–26.6	12.9	9.8–16.8
	C (%)	25.3	20.8–30.4	29.4	16.8–46.2	25.7	21.4–30.5
Treatment	ART (%)	89.8	86.2–92.5	95.3	84.6–98.7	90.4	87.1–92.9
Viral load	<40 cps/mL (%)	71.1	66.0–75.6	82.5	68.1–91.3	72.3	67.6–76.5
	< 1000 cps/mL (%)	86.3	82.2–89.5	87.5	73.9–94.5	86.4	82.6–89.5
CD4 count	Median (cells/μL)	468	129–807	490	173–807	468	134–803

IDU, injecting drug use; ART, antiretroviral therapy; NA, not applicable

^a^ Confidence interval 95% for proportions (Wilson)

^b^ Interquartile ranges

### HBV infection and vaccination status

According to their serological status, 37.6% of patients were naïve (susceptible) to HBV, 43.6% had a resolved infection, and 5.8% had chronic HBV infection (Table 3). There were no significant differences between chronically HBV-infected and not infected patients regarding age, gender, clinical categories, viral loads, and CD4 counts. Of the 23 patients with chronic HBV infection, 22 (96%) were on ART at time of inclusion. Of those, 21 (96%) received at least one HBV-active antiretroviral drug. Only 26 patients (6.5%) were classified as vaccinated (Table 3); of those, 10 (38%) had anti-HBs levels <100 IU/L, and 3 (12%) <10 IU/L. The proportion of vaccinated individuals was significantly lower (*p* = 0.0002) within the public health system (Table 3).

**Table 3:** Prevalence of HBV and HCV serological status of patients with HIV in Chile.

	Total (n = 399)			Fundación Arriarán (n = 353)			Clínica Alemana (n = 46)			Fisher Exact (two-tailed)
Serological status	N	%	CI95%	N	%	CI95%	n	%	CI95%	*p*
**Hepatitis B**										
Naïve (susceptible)	150	37.6	33.0–42.4	137	38.8	33.9–44.0	13	28.3	17.3–42.6	0.19
Vaccinated	26	6.5	4.5–9.4	16	4.5	2.8–7.2	10	21.7	12.8–37.0	0.0002
Resolved infection	174	43.6	38.8–48.5	153	43.3	38.3–48.6	21	45.7	32.2–59.8	0.87
Chronic infection	23	5.8	3.9–8.5	21	5.9	3.9–8.9	2	4.3	1.2–14.5	0.76
HBeAg negative	10	2.5	1.4–4.6	10	2.8	1.5–5.1	0	0.0	0–8.0	0.39
HBeAg positive	13	3.3	1.9–5.5	11	3.1	1.8–5.5	2	4.3	1.2–14.5	0.65
Isolated Anti-HBc	26	6.5	4.5–9.4	26	7.4	5.1–10.6	0	0.0	0–8.0	0.10
**Hepatitis C**										
Seropositive	4	1.0	0.4–2.6	3	0.8	0.3–2.2	1	0.3	0.04–1.4	0.39

CI95%, 95% confidence interval (Wilson interval without continuity correction)

### HCV infection

Four of the 399 patients (1%) were HCV infected (Table 3), three were male and the median age was 57.3 years (range 30.9 to 59.7 years). None of the patients had concurrent chronic HBV infection. Three of the four patients were from the public health center.

## Discussion

According to official national data, Chile has an HIV prevalence of 0.5% among the 15 to 49 year-old population [45]. Although classified as a low endemicity country, Chile is among the few countries with a recent increase of new HIV infections and mortality [1]. Chile is also a low endemicity country for HBV and HCV infection, with 0,15% and 0,01% seroprevalence, respectively, in the adult population (>15 years), according to the National Health Survey 2009–10 [46].

Epidemiological and clinical knowledge on coinfections with HBV and HCV of the Chilean HIV-infected population is scarce. A retrospective analysis of the Chilean AIDS Cohort database, which includes most public health centers in Chile, reported a coinfection rate of 8.4% (CI95% 7.3–9.8) with a higher, though not statistically significant mortality rate among those with HBV coinfection [22]. Another retrospective study of a single private HIV center in Santiago showed a prevalence of 6.1% (CI95% 4.0–9.3) of HBsAg carriers among 311 PLWH [21]. Both studies based solely on HBs antigen testing, which up to now is the only serological HBV parameter available in the routine care of PLWH in the public health system in Chile. The present study is the first cross-sectional study comparing coinfection rates among patients attending the public and private health services in Chile. The overall HBsAg-based rate was 5.8% (CI95% 3.9–8.5); it was lower in the private compared to the public sector (4.3% vs. 5.9%). The HIV/HBV coinfection rate in our population was in accordance with rates of other South American countries. In a recent review including data from Brazil, Argentina, Colombia, and Venezuela, the prevalence rates ranged from 1.9% to 8.5%, with the exception of two studies with higher rates [20]. The first study was from Buenos Aires and reported coinfection in14.5%, which most probably was due to the high percentage of IDU (48%) in the study population [36]. The second survey was from Brazil and had a rate of 10.5%; in this study, however, almost half of HBsAg positive individuals were anti-HBc negative, which is a very rare constellation, making the interpretation of the results difficult [47]. Studies from non-industrialized countries in Asia and Africa mostly report HIV/HBV coinfection in rates >10% [16], which might reflect on the higher overall endemicity of hepatitis B in those regions [48].

Among the HBsAg carriers of our study, more than half (57%) were HBeAg positive, indicating viral replication and infectivity. About half (50.1%) of all participants were anti-HBc positive; most of them together with anti-HBs, indicating past (resolved) HBV infection. This rate is slightly higher than the one found in a recent study from another Chilean region, where 42.7% of 192 HBsAg negative PLWH had anti-HBc antibodies [49]. A total of 26 (6.5%) of our population showed the serological pattern of “isolated anti-HBc” (anti-HBc positivity and anti HBs negativity). This constellation is more commonly detected in PLWH [50, 51]. As described in a recent review, “isolated anti-HBc” appears in 17%-40% of PLWH and is associated to HCV coinfection, older age, and elevated HIV RNA levels [52]. Our rate was much lower, but compatible with data from another Chilean survey [49].

The principal strategy to prevent Hepatitis B is vaccination, which is recommended for all HIV patients, but often not effectively implemented [53]. The Chilean public health policies endorse HBV vaccination since 1990. Initially only healthcare workers of hemodialysis units were included; later on, blood bank, laboratory, and emergency department staff [54]. In 2010, vaccine recommendations were expanded to other risk groups, but PLWH were not included. Hepatitis B as a routine childhood vaccine was implemented in 2005 in Chile and since then reached a high coverage of 95% [55]. HBV vaccinations is recommended in the Chilean HIV clinical guidelines, but without financial coverage is yet not guaranteed [56, 57]. Among our study population, only a minority was effectively vaccinated. This proportion was significantly lower in patients attending the public health system, suggesting financial barriers to access this intervention. On the other hand, a high percentage of all patients (28%) were identified as susceptible to HBV infection (thus requiring vaccination). Our data confirms the necessity to incorporate universal HBV vaccination for PLWH into the freely available HIV Program in Chile. Although this topic was addressed by the Chilean Ministry of Health in 2013, recommending vaccination of PLWH after testing for HBsAg and anti-HBc, no data on the coverage within the HIV population is available. In our experience, the requisite of serological testing for anti-HBc might be an obstacle for the implementation of this important preventive measure, since this exam is not covered by the public healthcare system. Further studies should be performed to determine if universal or targeted vaccination strategies are more cost-effective.

The efficacy of hepatitis B vaccines is affected by comorbidities and other factors such as obesity and active smoking [58, 59]. Whereas >90% of healthy adults develop protective antibodies after a 3-dose scheme [58], seroconversion rates for HIV positive patients only reach 47%; furthermore, the schedule is often not completed in HIV patients [60, 61]. In our study, more than one third of vaccinated patients had antibody concentrations <100 IU/L, requiring re-vaccination, and 12% had levels below the protection threshold of 10 IU/L. This is not surprising, since post-vaccination checks of anti-HBs levels, which are recommended in many countries [62, 63], are not routinely performed in Chile due to the high cost and lack of coverage by public or private health insurances.

With 1.0%, the HIV/HCV coinfection rate of our study population was very low, which is in accordance with previous data from Chile [21]. Similarly, low rates are reported from Colombia (0.8%) and Venezuela (0.7%). Other countries in Latin America shows higher coinfection rates (Argentina 29.9%, Brazil 18.9%, Cuba 12.9%, and Mexico 12.1%) [35]. In most other regions worldwide, HCV coinfection rates in PLWH reach much higher levels, e.g. 21% in USA [64] and 32.4% in Europe [65]. This is most probably associated to different prevalence rates of HCV-associated risks such as IDU and use of untested blood products. In Chile, IDU is very uncommon [66] and according to national data from 2012 to 2016, 99% of newly acquired HIV infections were sexually transmitted [45].

To our knowledge, our study is among the few that compare epidemiological and preventive aspects of PLWH within Chile’s two-tier health system. After 1973, the Military dictatorship promoted the development of private health insurances following aspects of market economy [67]. The coexistence of a public health insurance program (based on a social insurance model) with several market-based private health insurance companies is the main feature of the Chilean healthcare system. The fragmentation at insurance and provision levels produces two contrasting realities: an underfunded and overwhelmed public sector and increasingly expensive private sector [68, 69]. To date, the public system covers about 80–85% of the population, yet absorbs no more than 50% of active physicians [70]. In general, the public system provides less access to specialist services, has lower public expenditure and limited inpatient facilities, resulting in barriers to access to quality health services [70, 71]. For PLWH, these disparities have partly been reduced by the implementation of the Expanded Access Program in 2001 [3]. In our study, HBV exposure rates (resolved plus chronic infection) were similar in patients from both systems. For HCV, the prevalence tended to be higher in the public system, but numbers were too low to reach statistical significance. Thus, our study did not indicate relevant differences in the exposure to these two hepatic viruses among PLWH of the two healthcare systems in Chile. Still, the study demonstrated that the users of the public system were significantly less covered with the preventive measure of HBV vaccination.

A limitation of the study is that it only provides information from one region in Chile, the Metropolitan Region. Epidemiological data on HIV/HBV and HIV/HCV coinfections in other regions are not available. However, HBV and HCV rates of the general population are higher in the Metropolitan Region compared to most other regions in Chile [38], which might influence coinfection rates in PLWH. As demonstrated in southern Chile, ethnic differences might also contribute to differences in the epidemiology of HIV and other sexually transmitted diseases [72]. Due to economic reasons, our survey did not include molecular detection of HBV. This could have led to an underestimation of chronic hepatitis B cases, since some of them might present as “isolated anti-HBc” [52]. Finally, the recruitment for the study was not random, which could introduce bias. However, due to the high acceptance rate in the public hospital, we assume that it might not be significant. In the private health center, we were not able to reach the target number of participants within the study period. This might reflect the highly individualized patient care in this setting, where clinic-epidemiological studies are uncommon. In addition, not all patients had their routine laboratory tests performed at Clínica Alemana, and were therefore not available for the study.

In conclusion, this cross-sectional survey demonstrated a moderate level of HBV coinfection (5.8%) and a low level of HCV coinfection (1.0%) in PLWH from the Metropolitan Region in Chile. There was no significant difference of HBV or HCV infection in patients from the public versus the private health system, although studies with larger populations are necessary to confirm this finding. More than a third of patients were susceptible to hepatitis B, which shows that hepatitis B vaccination was not as widely used as internationally recommended. The rate of unvaccinated patients was significantly higher in the public system, highlighting that this preventive measure should urgently be reinforced in Chile and that overcoming health disparities remain a national challenge.
